# Invariant and Diverse NKT Cells Regulate Bacterial Clearance and Pathology in Chlamydial Genital Tract Infection in Mice

**DOI:** 10.3390/pathogens14111166

**Published:** 2025-11-15

**Authors:** Kazunari Ishii, Toshinori Soejima, Yoshiki Ohnishi, Ryo Ozuru, Ryota Itoh, Bin Chou, Michinobu Yoshimura, Akinori Shimizu, Yusuke Kurihara, Atsuhiko Sakamoto, Kenji Hiromatsu

**Affiliations:** 1 Department of Microbiology & Immunology, Faculty of Medicine, Fukuoka University, Fukuoka 814-0180, Japanozuru@fukuoka-u.ac.jp (R.O.); ryito@fukuoka-u.ac.jp (R.I.); choubin@fukuoka-u.ac.jp (B.C.); myoshimura@fukuoka-u.ac.jp (M.Y.); shimizua@fukuoka-u.ac.jp (A.S.); a.sakamoto.sw@fukuoka-u.ac.jp (A.S.); khiromatsu@fukuoka-u.ac.jp (K.H.); 2 Department of Otorhinolaryngology, Faculty of Medicine, Fukuoka University, Fukuoka 814-0180, Japan

**Keywords:** chlamydia, invariant NKT cells, diverse NKT cells, genital tract infection

## Abstract

*Chlamydia trachomatis* infection causes pelvic inflammatory disease and infertility, but how host immune factors control pathogen clearance or pathology is not fully understood. Using a mouse model of genital tract infection with *Chlamydia muridarum*, we examined the role of CD1d-restricted Natural killer T (NKT) cells. Invariant NKT cell-deficient mice (Jα18^−/−^) showed prolonged vaginal shedding of infectious elementary bodies (EBs), delayed clearance, and decreased early cytokine production compared to wild-type (WT) controls. Conversely, CD1d^−/−^ mice, which lack both invariant and diverse NKT cells, did not show significant differences in vaginal shedding compared to WT mice. Surprisingly, both NKT-deficient mice (Jα18^−/−^ and CD1d^−/−^) produced higher levels of inflammatory cytokines in the oviduct at day 35 post-infection (p.i.) and experienced more frequent upper genital tract pathology (hydrosalpinx) at day 80 p.i. However, no infectious EBs were recovered from the oviducts or uterine horns of NKT-deficient mice after day 35 p.i. Cortisone acetate reactivated infectious shedding in chronically infected NKT-deficient mice at day 100 p.i. These findings highlight distinct roles for NKT cell subsets: invariant NKT cells promote early clearance via rapid cytokine production, while the broader NKT population helps limit tissue damage. Targeting NKT pathways could help prevent chronic infection and infertility.

## 1. Introduction

Genital tract infection caused by *Chlamydia trachomatis* represents a significant global health concern, with an estimated 129 million new cases annually [1]. As an obligate intracellular bacterial pathogen, *C. trachomatis* relies heavily on host cellular processes for survival and replication within inclusion bodies [2]. The organism’s insidious nature and unique immune evasion mechanisms frequently lead to asymptomatic, persistent infections, which can, in turn, trigger severe inflammatory immunopathology [2,3]. Untreated infections frequently ascend to the upper female reproductive tract, causing pelvic inflammatory disease (PID), scarring, oviduct occlusion, and ultimately, infertility in 10–20% of cases [4]. The significant public health and economic impact highlights the urgent necessity for effective treatment and prevention strategies. Murine models of *Chlamydia muridarum* infection consistently mimic these key features of human *C. trachomatis* pathology, such as the development of hydrosalpinx and infertility, making them essential for studying host immune responses [5,6].

A crucial component of the immune system involved in recognizing lipid antigens is Natural Killer T (NKT) cells, a distinct subset of lymphocytes that bridge innate and adaptive immunity [7,8,9]. NKT cells recognize lipid and glycolipid antigens presented by the MHC class I-like molecule CD1d [10]. This broad category of T cells is divided into two main types: invariant NKT (type I NKT) cells and diverse NKT (type II) cells [10]. While invariant NKT cells are characterized by a semi-invariant T cell receptor (TCR) (Vα14-Jα18 in mice, Vα24-Jα18 in humans) and typically respond rapidly to specific antigens like α-galactosylceramide (α-GalCer) by producing a diverse array of cytokines, including gamma-interferon (IFN-γ), interleukin (IL)-4, and IL-13, diverse NKT cells possess a more diverse TCR and recognize different lipid antigens such as sulfatide [8,11,12]. NKT cells are activated during chlamydial infection when APCs present lipid antigens on CD1d [3,13].

Despite the recognized importance of NKT cells in other infectious settings, their specific and often complex, context-dependent roles in genital chlamydial infection remain under-investigated and ambiguous [3]. Previous studies have yielded mixed results, with invariant NKT cells demonstrating protection against respiratory *Chlamydia pneumoniae* by modulating dendritic cell (DC) function, primarily via increased IL-12 production by CD8α^+^DCs [14,15,16], but showing a more nuanced involvement in genital *C. muridarum* infection. For instance, Jiang J. et al. reported that the presence of CD1d-restricted NKT cells might attenuate chlamydial clearance and exacerbate immunopathology, as CD1d^−/−^ mice showed increased chlamydial vaginal burden and reduced oviduct immunopathology than wild-type (WT) mice [17]. On the other hand, Armitage et al. indicated that WT mice had significantly greater chlamydial genital burden than CD1d^−/−^ mice, and had significantly greater incidence and severity of upper genital tract immunopathology; however, invariant NKT-deficient mice (Jα18^−/−^ mice) had no significant differences in hydrosalpinx severity or incidence compared to WT controls, suggesting a minor role for invariant NKT cells in immunopathology [3]. The cytokine IL-17, often produced by invariant NKT cells [18], also exhibits a complex role in chlamydial infections; while IL-17 deficiency leads to reduced infection and pathology, it can also impair vaccine-induced protection [5]. This emphasizes the complex and sometimes conflicting roles of immune components in chlamydial diseases and the necessity for a better understanding of specific NKT cell subsets.

This study aimed to define the role of CD1d-restricted NKT cell subsets in modulating the progression and outcome of genital *C. muridarum* infection in mice. We demonstrate that invariant NKT cell deficiency prolongs genital shedding and delays pathogen clearance. Interestingly, CD1d^−/−^ mice lacking all NKT cells show less early shedding than invariant NKT-deficient (Jα18^−/−^) mice but develop more upper reproductive tract pathology, hydrosalpinx, later. These results indicate that invariant and diverse NKT cells have complex, sometimes opposing, effects on infection and pathology. Understanding these pathways may help develop treatments to accelerate clearance and reduce fibrosis and infertility.

## 2. Materials and Methods

### 2.1. Mice

Wild-type C57BL/6 mice (WT) were purchased from Japan SLC, Inc. (Hamamatsu, Shizuoka, Japan). Breeding pairs of CD1d^−/−^ mice, originally developed by Dr. Luc Van Kaer (Vanderbilt University), in the C57BL/6 background, and Jα18^−/−^ mice [19] with the C57BL/6 background, were provided by Dr. Yohichi Yasunami (Fukuoka University) [20]. CD1d^−/−^mice and Jα18^−/−^ mice were bred and maintained at the pathogen-free animal care facility (Fukuoka University, Fukuoka, Japan), and the mice used in the current study were 7- to 10-week-old female mice. All animal procedures and experimental protocols were approved by the Institutional Animal Care and Use Committee of Fukuoka University. Mice were housed in groups of no more than five per cage under controlled temperature (22 ± 2 °C), humidity (50 ± 10%), and a 12 h light/dark cycle, with ad libitum access to food and water. At the end of the experiment, **a**nimals were first placed under a deep plane of anesthesia to ensure humane euthanasia at the conclusion of the experiment. The anesthetic mixture, administered via the intraperitoneal route (i.p.), comprised medetomidine (Kyoritsu Seiyaku Corporation, Tokyo, Japan, 0.3 mg/kg), midazolam (Takeda Pharmaceutical Company Limited, Osaka, Japan, 4 mg/kg), and butorphanol (Meiji Seika Pharma Co., Ltd., Tokyo, Japan, 5 mg/kg). Following confirmation of non-responsiveness, euthanasia was performed by cervical dislocation. Mice were randomly assigned to three groups (*n* = 15/group at the start of the experiment). Due to unexpected animal attrition unrelated to the experimental procedures, the final group sizes for longitudinal analysis were 14 (WT mice), 9 (Jα18^−/−^ mice), and 10 (CD1d^−/−^ mice). All data were analyzed using these final numbers.

### 2.2. Microbes

*Chlamydia muridarum* (mouse pneumonitis strain Nigg II, ATCC VR-123) was obtained from the ATCC and propagated as previously described [21]. Chlamydial elementary bodies (EBs) were purified using Urografin (Bayer, Leverkusen, Germany) density gradient centrifugation, resuspended in sucrose-phosphate-glutamate (SPG) buffer, and stored at −80 °C. All Chlamydia stocks were confirmed negative for Mycoplasma contamination using a MycoAlert Mycoplasma detection kit (Lonza, Basel, Switzerland).

### 2.3. Intravaginal Infection of C. muridarum in Mice

Five days before infection and again at the time of infection, progesterone (Mochida Pharmaceutical Co., Ltd., Tokyo, Japan, 2.5 mg/mouse) was administered subcutaneously to synchronize the menstrual cycles and to increase the mouse susceptibility to chlamydial infection [22]. Mice were inoculated intravaginally with *C. muridarum* at 2 × 10^3^ inclusion-forming units (IFUs) in 5 μL of PBS.

### 2.4. IFU Assay

Mice were infected with *C. muridarum*, and vaginal secretions were collected with swabs on the indicated day. The swab head was immediately placed into a tube with 1.0 mL of SPG buffer (0.2 M sucrose, 0.02 M phosphate buffer, 5% fetal bovine serum, pH 7.2) and vortexed vigorously for 1 min. The suspension was stored frozen at −80 °C. Mice were euthanized on the indicated days after infection, perfused with 20 mL of EDTA (3.0 mg) containing PBS, and their genital organs were aseptically isolated and divided into three parts: cervix (Cx), uterine horn (UH), oviduct including ovary (Ov) [23]. These organs were homogenized in 2.0 mL of SPG buffer using a Model PRO 200 homogenizer (PRO Scientific Inc., Oxford, CT, USA). Then, the tissue homogenates were centrifuged at 1900× *g* for 30 min at 4 °C to remove coarse tissues and debris. The supernatants were collected and stored frozen at −80 °C. All *Chlamydia*-infected samples (vaginal secretion suspension and tissue homogenate supernatants) were collected, frozen, and thawed, then serially diluted 10-fold in SPG buffer, and 250 µL of the diluted sample was reseeded into 24-well plates containing a monolayer of HeLa cells (2 × 10^5^ cells per well). The plates were centrifuged at 900× *g* for 60 min, then washed and cultured in DMEM with 5% FCS and 1 μg/mL cycloheximide. After 24–48 h, the cells were fixed with ice-cold methanol and incubated with an FITC-conjugated anti-Chlamydia LPS-specific monoclonal antibody (PROGEN, Heidelberg, Germany) and DAPI. The growing chlamydial inclusions were counted using Axioskop fluorescent microscopy (Carl Zeiss, Oberkochen, Germany). Finally, the mean number of IFUs was calculated. The limit of detection for this assay was calculated as 4 IFU per vaginal swab and 8 IFU per organ. Results below these thresholds were considered below the limit of detection.

### 2.5. Cytokine ELISA

Swab samples were collected as described in the IFU section. Genital organs were dissected in the same manner. A 0.5 mL aliquot of SPG buffer was added to the dissected oviduct tissue, which was then ground on a stainless-steel mesh to prepare samples for ELISA. The concentrations of mouse MIP-2, IL-6, KC, IL-1β, IL-1*α*, TNF-*α*, IL-12p70, IFN-*γ*, IL-17A, and IL-12p40 in the vaginal swabs and ovarian samples were measured using the DuoSet ELISA development system (R&D Systems Inc., Minneapolis, MN, USA) according to the manufacturer’s instructions. The absorbance of the ELISA plates was read using a Model 680 microplate reader (BIO-RAD Laboratories, Hercules, CA, USA).

### 2.6. Assessment of Upper Genital Tract Pathology

Mice were sacrificed 80 days post-infection and the genital tract tissues were removed. At the macroscopic level, upper genital tract pathology was determined for evidence of hydrosalpinx formation [24]. The oviduct sections were stained with hematoxylin and eosin (H&E), and the maximum oviduct diameter of each mouse was measured using the KEYENCE BZ-II analyzer (KEYENCE CORPORATION, Osaka, Japan, Ver. 1.42).

### 2.7. Immunohistochemistry

Genital organs were isolated from mice on the specified days post-infection. The tissue was fixed in 4% paraformaldehyde, embedded in paraffin blocks, and sectioned. Tissue sections were deparaffinized and rehydrated. After antigen retrieval and quenching endogenous peroxidases, the sections were blocked with 10% goat serum (Cedarlane Laboratories Ltd., Burlington, ON, Canada) in PBS for 1 h at room temperature. *C. muridarum* antigens were detected using a rabbit polyclonal anti-*Chlamydia* antibody (Abcam, Cambridge, UK) and an EnVision + Kit/HRP secondary antibody (Dako, Agilent Technologies, Inc., Santa Clara, CA, USA). All sections were developed with diaminobenzidine, washed, and counterstained with hematoxylin. Negative controls without primary antibodies were processed the same way. Images were captured using a BZ-9000 digital CCD microscope (KEYENCE).

### 2.8. Real-Time Quantitative Reverse Transcription-PCR (qPCR)

Oviduct genital organs were harvested, and Total RNA was extracted using ISOGEN II (NIPPON GENE, Tokyo, Japan) according to the manufacturer’s instructions. First-strand cDNA synthesis was performed using total RNA and the SuperScript^®^ III First Strand Synthesis System (Thermo Fisher Scientific, Carlsbad, CA, USA). The resulting cDNA was used for qPCR. qPCR was conducted using the 7500 Real-Time PCR system (Applied Biosystems, Inc., Waltham, MA, USA) and SYBR^®^ Premix DimerEraser™ (TaKaRa Bio Inc., Shiga, Japan). The thermal cycling conditions were: initial denaturation at 95 °C for 30 s, followed by 40 cycles of denaturation at 95 °C for 5 s, annealing at 55 °C for 30 s, and extension at 72 °C for 34 s. The relative expression level of *C. muridarum* hsp60 mRNA was calculated by normalizing the expression to that of the mouse reference gene β-actin (MmActb) using the 2^−ΔΔCt^ method. The primer sequences used were as follows: *C. muridarum* hsp60 (Cmhsp60):Fw: 5′-AGGACTAGGCGGGGTTGTAT-3′ Rv: 5′-TGGGGCAGCTCTTCTTTACG-3′ Mouse β-actin (MmActb):Fw: 5′-CACTGTCGAGTCGCGTCC-3′ Rv: 5′-TCATCCATGGCGAACTGGTG-3′.

### 2.9. Cortisone Acetate Treatment

The experiments of reactivation of chlamydial infection by immunosuppression were performed according to the method described by Cotter T. W. et al. [25]. Mice more than 100 days post-infection received cortisone acetate (FUJIFILM Wako, Osaka, Japan) daily for 10 consecutive days. The daily dose of cortisone acetate was 3.1 mg (125 mg/kg) in 0.2 mL of saline administered intraperitoneally. To evaluate *C. muridarum* reactivation, vaginal swabs were collected at 4, 7, 10, and 14 days following the initiation of immunosuppression and frozen at −80 °C. Reactivation of chlamydial shedding was assessed by IFU assay using cultured HeLa cells.

### 2.10. Statistical Analysis

Continuous data are expressed as the mean ± SD. For analysis of multiple groups, one-way or two-way ANOVA was performed, followed by Tukey’s post hoc test for multiple comparisons. Comparisons of Area Under the Curve (AUC) values among groups were performed using the Kruskal–Wallis test, followed by Dunn’s multiple comparisons test, controlling the False Discovery Rate using the original method of Benjamini and Hochberg. These analyses were performed using GraphPad Prism software (GraphPad, La Jolla, CA, USA, version 9.5.1). The time to bacterial clearance was estimated using the Kaplan–Meier method, and curves were compared using the log-rank test. When a significant difference was detected, pairwise comparisons were conducted using the log-rank test with the Bonferroni correction, performed using R software (version 4.4.0). Frequencies of hydrosalpinx were compared across WT, Jα18^−/−^, and CD1d^−/−^ groups using a chi-squared test, followed by pairwise Fisher’s exact tests with Bonferroni correction for post hoc analysis. All statistical tests were two-sided, and a *p*-value of less than 0.05 was considered statistically significant. Significance levels are denoted as follows: * *p* < 0.05, ** *p* < 0.01, *** *p* < 0.001, and **** *p* < 0.0001. For the primary endpoints (bacterial clearance time and hydrosalpinx incidence), effect sizes were calculated to assess the magnitude of the differences (Cohen’s d for continuous data and Cohen’s h for proportional data). A retrospective power analysis was also performed for these endpoints, with details provided in the Supplementary Materials (see Supplementary Note S1).

## 3. Results

### 3.1. Invariant NKT Cell-Deficient Mice (Jα18^−/−^Mice) Exhibit Prolonged Shedding of Infectious Elementary Bodies (EB)

**Figure 1:**
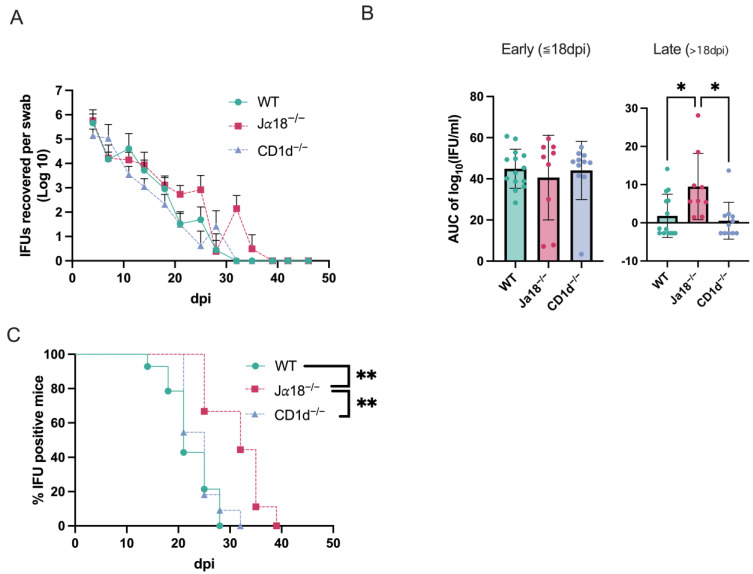
Invariant NKT cell-deficient mice (Jα18^−/−^ mice) exhibit extended shedding of infectious elementary bodies (EBs). WT mice, Jα18^-/-^ mice, CD1d^−/−^ mice were intravaginally infected with 2 × 10^3^ IFUs of *C. muridarum*, and vaginal swabs were collected at 3–4-day intervals. Infectious EB in swabs was measured by IFU assay in cultured HeLa cells (WT *n* = 14, Jα18^−/−^ *n* = 9, CD1d^-/-^ *n* = 10). The detection limit for the IFU assay was established at 4 IFU per vaginal swab. Negative results indicate titers below this threshold. Swab average IFUs ± SD over time for each group are shown (**A**), followed by Area under the curve (AUC) analysis (**B**). Dots represent individual data points. The percentage of mice with positive *Chlamydia* culture for each group is shown (**C**). Statistical analyses were performed using a Kruskal–Wallis test followed by Dunn’s multiple comparisons test, controlling the False Discovery Rate using the original method of Benjamini and Hochberg (**B**) and the log-rank test with the Bonferroni correction (**C**). * *p* < 0.05, ** *p* < 0.01.

We investigated whether CD1d-restricted invariant NKT cells or CD1d-restricted NKT cells (diverse NKT and invariant NKT) could play a role in host defense against chlamydial genital tract infection and its pathogenesis. Groups of mice (C57BL/6 WT mice: *n* = 14, C57BL/6 background Jα18^−/−^ mice: *n* = 9, and C57BL/6 background CD1d^−/−^ mice: *n* = 10) were intravaginally infected with *C. muridarum*, and vaginal shedding of live organisms was monitored throughout the infection using IFU assay on samples collected via vaginal swabs. The absence of invariant NKT cells caused a slight but significant delay in the resolution of infection and a significant increase in bacterial burden during the infection compared to WT controls or CD1d^−/−^ mice (Figure 1). All groups of mice (WT, Jα18^−/−^, and CD1d^−/−^ mice) shed similar numbers of infectious elementary bodies (EB) into the vagina during the first week (day 4 and 7 post-infection). The mean IFU counts of vaginal swabs from CD1d^−/−^ mice on days 11, 14, 18, and 25 post-infection were lower than those from WT mice but did not reach statistical significance. Jα18^−/−^ mice showed delayed bacterial clearance, with chlamydial infectious EB shedding persisting until day 35 (Figure 1A). Area under the curve (AUC) analysis confirms increased infection in Jα18^−/−^ mice during the late phase of infection (<day 18) (Figure 1B). The percentage of mice with swabs containing IFU-positive results among WT, Jα18^−/−^, and CD1d^−/−^ mice indicated that invariant NKT cell-deficient mice (Jα18^−/−^) have an extended period of infectious EB shedding (*p* < 0.01; Cohen’s d = 1.94, 95% CI [0.90, 2.94]) (Figure 1C). These results suggest increased susceptibility to *C. muridarum* genital tract infection in invariant NKT-deficient mice.

### 3.2. Mice Without Invariant NKT Cells Produce Significantly Fewer Inflammatory Cytokines in Vaginal Swabs During the Early Stage of Intravaginal Chlamydial Infection

**Figure 2:**
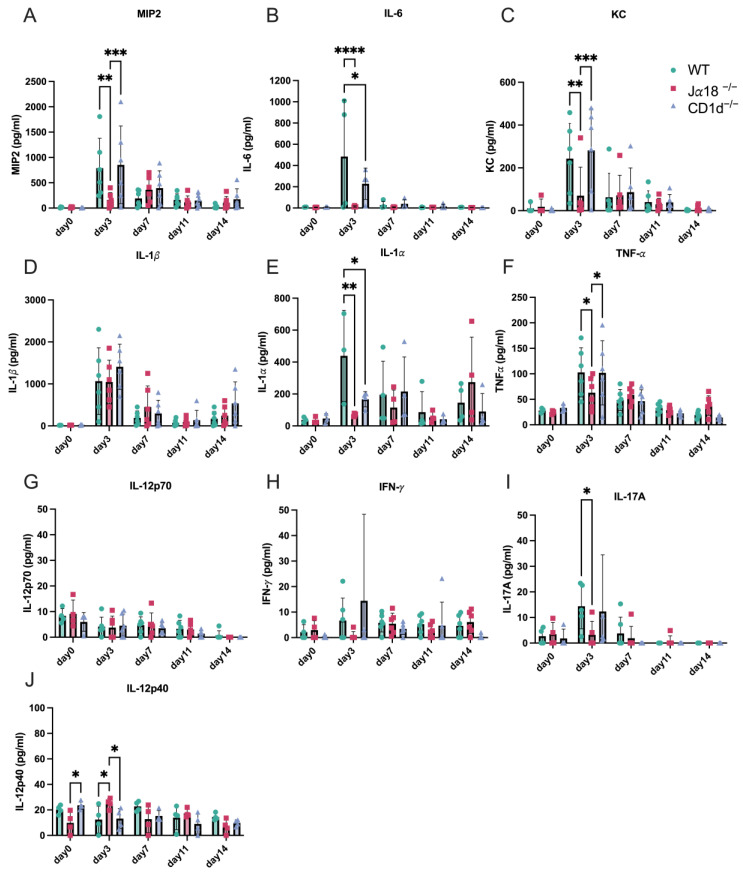
Invariant NKT cell-deficient mice (Jα18^−/−^) produce fewer inflammatory cytokines in vaginal swabs early in chlamydia infection than WT and/or CD1d^−/−^ mice. Levels of MIP2 (**A**), IL-6 (**B**), KC (**C**), IL-1β (**D**), IL-1*α* (**E**), TNF-α (**F**), IL-12p70 (**G**), IFN-*γ* (**H**), IL-17A (**I**), and IL-12p40 (**J**) from vaginal swab samples were measured in WT, Jα18^−/−^, and CD1d^−/−^ mice during *C. muridarum* intravaginal infection on days 0, 3, 7, 11, and 14 post-infection. Statistical analyses were performed using a two-way ANOVA followed by Tukey’s multiple comparison test (**A**–**J**). * *p* < 0.05, ** *p* < 0.01, *** *p* < 0.001, and **** *p* < 0.0001.

Next, we measured cytokine levels in vaginal swabs from WT, Jα18^−/−^ mice, and CD1d^−/−^ mice after intravaginal infection with *C. muridarum*. The peak production of MIP-2 (also known as CXCL2), IL-6, KC (also known as CXCL1), IL-1β, TNFα, and IL-17A occurred on day 3 in WT and CD1d^−/−^ mice (Figure 2). In contrast, invariant NKT-deficient mice (Jα18^−/−^ mice) did not produce significant levels of these early inflammatory cytokines, and MIP-2, IL-6, KC, IL-1α, TNFα, and IL-17A levels on day 3 p.i. from Jα18^−/−^ mice were significantly lower compared to those in WT and/or CD1d^−/−^ mice (Figure 2). These findings align with the delay in bacterial clearance observed in Jα18^−/−^ mice (Figure 1B,C).

### 3.3. Invariant NKT-Deficient Mice (Jα18^−/−^) and Mice Lacking Both Invariant and Diverse NKT Cells (CD1d^−/−^) Produce Higher Levels of Inflammatory Cytokines in the Oviducts During the Late Stage of Chlamydial Intravaginal Infection

**Figure 3:**
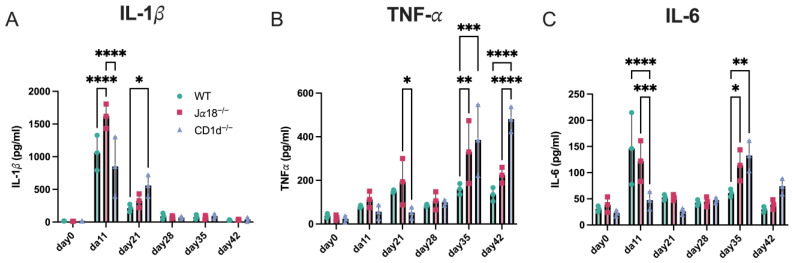
Effect of invariant NKT cell deficiency or NKT (diverse and invariant NKT) cell deficiency on inflammatory cytokines in oviduct tissues after *C. muridarum* intravaginal infection. Levels of IL-1β (**A**), TNF-α (**B**), and IL-6 (**C**) were measured in tissue homogenates from the oviduct including ovary of WT, Jα18^−/−^, and CD1d^−/−^ mice. Cytokine levels were assessed on days 0, 11, 21, 28, 35, and 42 following intravaginal infection with *C. muridarum*. *n* = 3 mice per group at each time point. Statistical analyses were performed using a two-way ANOVA followed by Tukey’s multiple comparison test (**A**–**C**). * *p* < 0.05, ** *p* < 0.01, *** *p* < 0.001, and **** *p* < 0.0001.

It is well known that *C. trachomatis* infection in the female genital tract can ascend to the upper genital areas, leading to hydrosalpinx, a major sign of tubal infertility in women. Consequently, we examined how inflammatory cytokine levels changed in the upper genital tract of infected mice from day 11 to day 35 post-infection. Levels of IL-1β, TNF-α, and IL-6 in tissue homogenates from the oviduct, including the ovary, were measured in WT, Jα18^−/−^, and CD1d^−/−^mice on days 11, 21, 35, and 42 after *C. muridarum* intravaginal infection (Figure 3). It is worth noting that IL-1β and IL-6 levels on day 11 in the lysates of oviducts from Jα18^−/−^ mice were significantly higher than those in WT or CD1d^−/−^ mice, which might reflect an increased bacterial burden in Jα18^−/−^ mice (Figure 1). Surprisingly, we observed significant increases in TNF-α on day 35 p.i. and day 42 p.i., and IL-6 on day 35 p.i. in oviduct lysates from Jα18^−/−^ and CD1d^−/−^ mice compared to WT mice. These results suggest that the absence of invariant NKT cells (Jα18^−/−^ mice) or the lack of both invariant and diverse NKT cells (CD1d^−/−^ mice) may promote immunopathology in the upper genital tract during the late stage of *C. muridarum* infection.

### 3.4. NKT Cell Deficiency Raises the Risk of Upper Genital Tract Pathology (Hydrosalpinx) After Intravaginal Infection with C. muridarum

**Figure 4:**
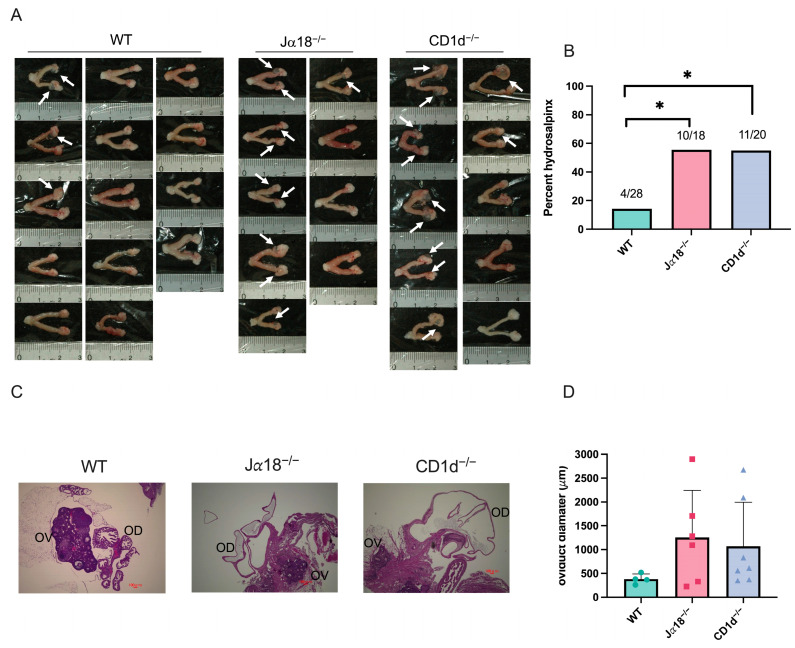
NKT cell deficiency raises the risk of upper genital tract pathology (hydrosalpinx) after intravaginal infection with *C. muridarum*. Female reproductive tissues from WT, Jα18^−/−^, and CD1d^−/−^ mice infected with 2 × 10^3^ IFU of *C. muridarum* were assessed at day 80 post-infection. (**A**) Hydrosalpinx occurrence was observed macroscopically. Arrowheads show hydrosalpinx. The numbers in the figure indicate the actual number of hydrosalpinx cases in the total number of oviducts (WT *n* = 28, Jα18^−/−^ *n* = 18, CD1d^−/−^ *n* = 20). (**B**) The graph shows the incidence of hydrosalpinx in each mouse group. (**C**) H&E-stained paraffin-embedded sections of mouse reproductive organs 80 days post-infection. OV: ovary; OD: oviduct. Scale bar: 100 μm. (**D**) Oviduct diameter was measured in each mouse group using KEYENCE BZ-II analyzer. Circles, squares, and triangles represent individual data points for the WT, Jα18^−/−^, and CD1d^−/−^ groups, respectively. Statistical analyses were performed using a chi-squared test followed by pairwise Fisher’s exact tests with Bonferroni correction (**B**), and a one-way ANOVA followed by Tukey’s multiple comparison test (**D**). * *p* < 0.05.

Next, we investigated whether invariant NKT cells and/or diverse NKT cells affect upper genital tract pathology, especially hydrosalpinx, following intravaginal infection with *C. muridarum* in mice. When comparing the gross pathology of genital tract tissues collected 80 days after infection among three groups of mice (WT, Jα18^−/−^ mice, and CD1d^−/−^ mice), we observed that 4 out of 28 oviducts from WT mice, 10 out of 18 oviducts from Jα18^−/−^ mice, and 11 out of 20 oviducts from CD1d^−/−^ mice developed hydrosalpinx (Figure 4A). The occurrence of hydrosalpinx caused by *C. muridarum* infection was significantly higher in NKT-deficient mice (Jα18^−/−^ and CD1d^−/−^ mice) compared to WT mice (*p* < 0.05, Fisher’s exact test; Cohen’s h = 0.91) (Figure 4B). When H&E staining of genital tract tissues was examined under a microscope, Jα18^−/−^ and CD1d^−/−^ mice also showed more severe oviduct luminal dilation, although this did not reach a significant difference (Figure 4C,D). These findings show that lacking invariant NKT cells (Jα18^−/−^ mice) or both invariant and diverse NKT cells (CD1d^−/−^ mice) raises the risk of fallopian tube oviduct pathology after *C. muridarum* infection.

### 3.5. Immunosuppression with Cortisone Acetate Reactivates the Persistent Infection of C. muridarum in NKT-Deficient Mice

**Figure 5:**
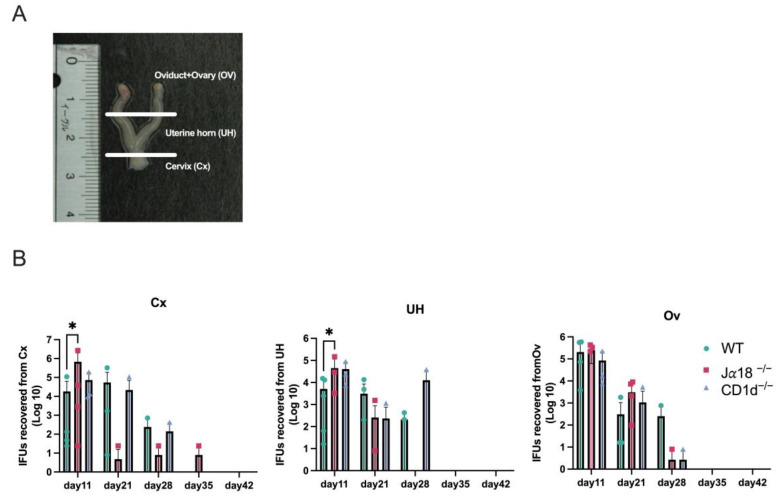
Mouse reproductive organ IFU assays following *C. muridarum* infection. Homogenates from the cervix (Cx), uterine horns (UH), and oviducts (OD) of WT, Jα18^−/−^, and CDId^−/−^ mice were subjected to IFU assays on days 11, 21, 28, 35, and 42 post-infection. (**A**) The genital organs were divided into three parts: the cervix, uterine horns, and oviducts including ovaries. (**B**) Each section was homogenized for use in the IFU assay. Statistical analyses were performed using a two-way ANOVA followed by Tukey’s multiple comparison test (**B**). The detection limit for the IFU assay was established at 8 IFU per organ. Negative results indicate titers below this threshold. * *p* < 0.05.

**Figure 6:**
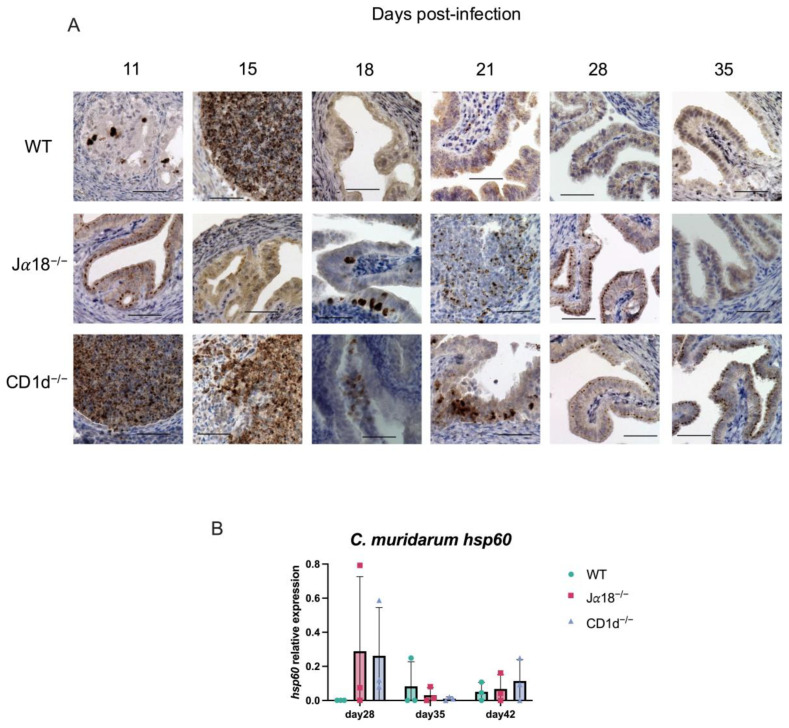
Immunohistochemical detection of chlamydial antigen in the oviducts from WT, Jα18^−/−^, and CD1d^−/−^ mice. Oviducts from *C. muridarum*-infected mice were harvested on the days indicated in the figure (days 11, 15, 18, 21, 28, and 35 post-infection). Formalin-fixed paraffin sections were stained by reacting with a rabbit polyclonal anti-*Chlamydia* antibody and an EnVision+ anti-rabbit antibody. The antigens were detected using DAB as the substrate. The figure shows representative immunohistochemical staining images for each group of mice on each day. (**A**) ×400; scale bar, 50 μm. (**B**) The bacterial load in the oviducts of infected mice (WT, Jα18^−/−^, and CD1d^−/−^) was quantified by real-time quantitative reverse transcription-PCR (qPCR) targeting the *C. muridarum* hsp60 mRNA. Data are shown as the mean ± SD (*n* = 3 mice per group). Statistical significance was determined by two-way ANOVA followed by Tukey’s multiple comparison test.

Although we observed significant increases in TNF-α on days 35 and 42 p.i. and IL-6 on day 35 p.i. in oviduct lysates from Jα18^−/−^ and CD1d^−/−^ mice, we rarely recovered infectious EBs from vaginal swabs after day 35 p.i. We also did not recover infectious EBs from upper genital tract tissues such as OV (Oviducts + Ovary) and UH (uterine horn), obtained from Jα18^−/−^, CD1d^−/−^, and WT mice starting from day 35 p.i. (Figure 5). We also performed an immunohistochemical study to detect chlamydial antigens in the oviducts of mice on days 11, 15, 18, 21, 28, and 35 p.i. Chlamydial inclusion staining in the oviducts of WT mice was observed on days 11 and 15 p.i., but it decreased significantly after day 18 p.i., whereas in Jα18^−/−^ and CD1d^−/−^ mice, chlamydial inclusion staining persisted beyond day 18 and up to day 35 p.i. (Figure 6A). Chlamydial 60 kDa heat-shock protein (hsp60) is well recognized as a persistent infection-associated antigen [26]. Real-time RT-PCR also revealed that chlamydial hsp60 mRNA expression was upregulated in oviducts of Jα18^−/−^ and CD1d^−/−^ at day 28 p.i. and later (Figure 6B). Taken together, these findings suggest that persistent, rather than proliferative, chlamydial infection may be occurring in the upper genital tract of NKT-deficient mice (Jα18^−/−^ and CD1d^−/−^) during the late stage of *C. muridarum* infection in the genital tract.

To further examine the above possibility of persistent chlamydial infection in NKT-deficient mice, we induced immunosuppression in chronically infected mice by administering corticosteroid hormone. Mice more than 100 days post-infection received cortisone acetate (3.1 mg per mouse) daily for 10 days straight. To assess *Chlamydia* reactivation, vaginal swabs were taken at 4, 7, 10, and 14 days after the start of cortisone treatment. The presence of infectious elementary bodies (EBs) in the swabs was measured by counting inclusion-forming units (IFUs) using cultured HeLa cells as the detection method. We found that cortisone acetate-induced immunosuppression reactivated chlamydial infection in NKT-deficient mice (Jα18^−/−^and CD1d^−/−^ mice) (Table 1).

## 4. Discussion

Our findings demonstrate a critical role for CD1d-restricted natural killer T (NKT) cells and, in particular, invariant NKT cells, in regulating both the resolution of *C. muridarum* genital tract infection and the associated immunopathology. The absence of invariant NKT cells (Jα18^−/−^ mice) resulted in prolonged vaginal shedding of infectious elementary bodies (EBs), delayed clearance, and a higher bacterial burden compared to WT mice. In contrast, CD1d^−/−^ mice, which lack both invariant and diverse NKT cells, did not show significant differences in vaginal shedding compared to WT mice, but still marked pathology in the upper genital tract at the late phase of infection. Taken together, these results suggest a differential and complex contribution of distinct NKT subpopulations in host defense and disease pathogenesis during chlamydial infection.

The impaired ability of Jα18^−/−^ mice to produce early inflammatory cytokines and chemokines such as MIP-2, IL-6, KC, IL-1α, TNF-α, and IL-17 following intravaginal inoculation indicates that invariant NKT cells are important contributors to the early inflammatory milieu. These cytokines are well established as critical mediators for neutrophil recruitment and for shaping the subsequent adaptive immune response. Qiao et al. reported that endogenous IL-17 mediates neutrophil infiltration by promoting chemokines such as MIP-2, KC, and IL-6 during *C. muridarum* lung infection in mice [27]. Multiple subsets of invariant NKT cells have been identified, including IL-17-producing invariant NKT cells, which are commonly called NKT17 cells [18]. Therefore, it is possible to speculate that invariant NKT cells, which are activated during *C. muridarum* infection, secrete IL-17 and induce neutrophil-related cytokine production. The early cytokine deficiency observed in invariant NKT-deficient mice (Jα18^−/−^ mice) correlates with higher bacterial burden and slower clearance, supporting the idea that invariant NKT-derived signals quickly act to contain infection in the lower genital tract. These findings align with previous studies indicating that invariant NKT-derived IFN-γ and IL-17 can be crucial in restricting intracellular pathogens [8]. Interestingly, although CD1d^−/−^ mice also lack invariant NKT cells, their vaginal IFU burden was not increased and even trended lower than WT controls, suggesting that other CD1d-restricted non-invariant NKT subsets may actually promote bacterial replication or influence host responses differently than invariant NKT cells. This difference highlights the distinct and sometimes opposing roles of invariant versus diverse NKT cell subpopulations in bacterial infection. On the other hand, Armitage et al. reported that BALB/c CD1d^−/−^ mice (NKT-deficient) had a significantly lower chlamydial burden and less upper genital tract pathology compared to control BALB/c WT mice after *C. muridarum* genital tract infection, concluding that non-invariant NKT cells (Type II NKT cells) play a harmful or immunopathogenic role [3]. This was inferred because CD1d^−/−^ mice showed less pathology, whereas C57BL/6 Jα18^−/−^ mice (which lack only invariant NKT cells) did not exhibit reduced pathology compared to C57BL/6 WT mice [3]. The difference in mouse strain (BALB/c vs. C57BL/6) and precise experimental protocols could contribute to the discrepancies observed between these studies. These differences emphasize the need to carefully consider experimental models.

Both Jα18^−/−^ and CD1d^−/−^ mice exhibited increased inflammatory cytokine production in the oviducts at day 35 and a higher incidence of hydrosalpinx compared to WT mice at day 80 p.i. These results suggest that while invariant NKT cells assist in early clearance in the lower genital tract, a broader NKT cell population—including both invariant and diverse NKT cells—is essential to prevent upper genital tract pathology during the later stage of chlamydial genital tract infection. The failure to detect recoverable infectious EBs via IFU assay, despite elevated inflammatory cytokine levels in the oviducts and the presence of chlamydial antigen in oviducts, indicates a persistent, non-replicating state within the genital tract epithelium at this advanced stage. Therefore, the increased pathology seen in NKT-deficient mice may stem not only from unrestrained bacterial growth early in infection but also from a sustained inflammatory response to persistent chlamydial antigen reservoirs.

Our cortisone acetate experiments raise the possibility that the latent (persistent) chlamydial infection in NKT-deficient mice can be reactivated, resulting in recoverable infectious EBs even more than 100 days after infection. This clearly confirms that viable, although dormant, *Chlamydia* persists long-term in the genital tract of these animals [3]. This issue is significant because the continued presence and possible reactivation of chlamydial infection are thought to play a role in the recurrent and persistent manifestation of genital chlamydial disease in humans. Our data directly support a mechanistic link between NKT cell deficiencies, impaired infection resolution, and subsequent persistence with pathogenic effects.

In human infection with *C. trachomatis*, delayed clearance and chronic persistence are major risk factors for pelvic inflammatory disease, ectopic pregnancy, and infertility [28,29]. Our results suggest that invariant NKT cells may play a protective role by promoting early cytokine responses and bacterial clearance, whereas NKT populations as a whole may balance host defense against immunopathology. Deficits in these cell subsets could therefore predispose to chronic infection and tubal damage in women. The apparent dichotomy of invariant NKT versus diverse NKT roles may help explain variability in infection outcomes among individuals and represents an avenue for further immunological investigation.

While our findings offer new insights into the specific functions of NKT cell subsets during chlamydial infection, we recognize several limitations that must be taken into account when interpreting these results. First, the differences in vaginal shedding or pathology between the wild-type and KOs are not very obvious, although consistent differences were observed. We acknowledge the limited differences seen and suggest that future studies, exploring different time points or infection doses, could better clarify the subtle yet important role of NKT cells in this infectious model. Second, due to unexpected animal attrition unrelated to the experimental procedures, the group sizes for some of our longitudinal analyses became unbalanced. Although our retrospective power analysis (Supplementary Note S1) indicates that the study was sufficiently powered for our primary endpoints, future studies with larger, balanced cohorts would improve the robustness of our conclusions. Third, some of our secondary analyses were limited by small sample sizes. Specifically, the analysis of late-stage oviduct cytokines was conducted with a small number of samples, which might reduce the ability to detect subtle differences and could be affected by individual variability. Similarly, in the cortisone-induced reactivation experiment, the lack of reactivation in the wild-type group should be interpreted cautiously. This observation might be a type II statistical error caused by under-sampling, and this negative result warrants further validation in a larger study. Furthermore, our retrospective power analysis showed that the study was underpowered to detect the small-to-moderate difference in bacterial clearance time observed between WT and CD1d^−/−^ mice (power = 0.17). As a result, we cannot definitively rule out a modest biological effect in this comparison, and the finding should be viewed with caution. Despite these limitations, this study offers a solid foundation and points out key areas for future research. Future work using larger animal cohorts and complementary methods, such as antibody-mediated cell depletion, will be helpful to further understand the specific mechanisms identified here.

## 5. Conclusions

Our work positions NKT cells at the intersection of pathogen clearance and host pathology during chlamydial infection. By demonstrating that distinct NKT subsets exert nonredundant, even divergent, effects, this study advances the conceptual framework of immunoregulation in persistent bacterial infections. Further dissection of NKT-driven pathways could reveal therapeutic targets to accelerate clearance while limiting fibrosis and infertility associated with chronic chlamydial infection.

## Figures and Tables

**Table 1:** Cortisone acetate-induced immunosuppression reactivated chlamydial infection in NKT-deficient mice. Mice more than 100 days post-infection received cortisone acetate (3.1 mg per mouse) daily for 10 consecutive days. To evaluate *C. muridarum* reactivation, vaginal swabs were collected at 4, 7, 10, and 14 days after starting cortisone treatment. The presence of infectious elementary bodies (EBs) in the swabs was determined by counting inclusion-forming units (IFUs) using cultured HeLa cells. Percentages of mice showing infectious EBs shedding in vaginal swabs (reactivation of *C. muridarum* infection) determined by IFU assays are displayed. The number in parentheses indicates the actual count of mice. For example, 3/13 means 3 mice out of 13 mice showed reactivation of *C. muridarum*. The detection limit for the IFU assay was established at 4 IFU per vaginal swab. Negative results indicate titers below this threshold.

Mice	% of Animals Reactivated for MoPn Shedding
WT	0 (0/9)
J*α*18^−/−^	23 (4/13)
CD1d^−/−^	7 (1/15)

## Data Availability

The original contributions presented in the study are included in the article, and further inquiries can be directed to the corresponding author.

## Supplementary Materials

The following supporting information can be downloaded at: https://www.mdpi.com/article/10.3390/pathogens14111166/s1. Supplementary Note S1: Retrospective Power Analysis.
