# Supplementary material for: Invariant and Diverse NKT Cells Regulate Bacterial Clearance and Pathology in Chlamydial Genital Tract Infection in Mice
Source: Pathogens. 2025 Nov 15;14(11):1166. doi: 10.3390/pathogens14111166 (PMC12655205; doi:10.3390/pathogens14111166)
Supplement: Supplementary file 1 [file pathogens-14-01166-s001.zip › pathogens-3882812-supplementary.pdf]

Supplementary Note 1: Retrospective Power Analysis

To address the potential concern about the differences in sample sizes among the mouse groups (WT mice n=14, Jα18$^{-/-}$ mice n=9, CD1d$^{-/-}$ mice n=10) in Figures 1 and 4, we performed a retrospective power analysis for the study's two main endpoints: (1) time to bacterial clearance and (2) incidence of hydrosalpinx. The analysis was conducted to assess whether the sample sizes were sufficient to detect the observed differences between key experimental groups.

Methods:

Power calculations were performed using the pwr package in R (version 4.4.0). For the continuous endpoint (time-to-clearance), power was calculated for a two-sample, two-sided t-test with unbalanced sample sizes (pwr.t2n.test), using Cohen's d as the effect size. For the categorical endpoint (hydrosalpinx incidence), power was calculated for a two-proportion test with unbalanced sample sizes (pwr.2p2n.test), using Cohen's h as the effect size. For the hydrosalpinx analysis, and consistent with the presentation in Figure 4, each oviduct was treated as an individual sample. The significance level (α) was set at 0.05 for all analyses.

Results:

1. Power Analysis for Bacterial Clearance Time

- **WT vs. Jα18-/- Comparison:** With sample sizes of n=14 (WT) and n=9 (Jα18$^{-/-}$), the observed effect size was very large (Cohen's d = 1.94). The resulting statistical power was 0.99 (99%).
- **WT vs. CD1d-/- Comparison:** With sample sizes of n=14 (WT) and n=11 (CD1d$^{-/-}$), the observed effect size was small-to-moderate (Cohen's d = 0.42). The resulting statistical power was 0.17 (17%).

2. Power Analysis for Hydrosalpinx Incidence

- **WT vs. Jα18$^{-/-}$ Comparison:** With sample sizes of n=28 (WT oviducts) and n=18 (Jα18$^{-/-}$ oviducts), the observed effect size was large (Cohen's h = 0.91). The resulting statistical power was 0.85 (85%).
- **WT vs. CD1d-/- Comparison:** With sample sizes of n=28 (WT oviducts) and

n=20 (CD1d$^{-/-}$ oviducts), the observed effect size was large (Cohen's h = 0.90). The resulting statistical power was 0.86 (86%).

Overall Conclusion:

This analysis confirms that our study had sufficient statistical power to draw robust conclusions for the majority of its primary findings. This includes the delayed bacterial clearance in Jα18$^{-/-}$ mice and the increased incidence of hydrosalpinx in both Jα18$^{-/-}$ and CD1d$^{-/-}$ mice compared to WT controls. The analysis also highlights that the study was underpowered to definitively assess differences in clearance time between the WT and CD1d$^{-/-}$groups; therefore, this specific finding should be interpreted with caution, as noted in the main text.
